# The Position of Sailors and Soldiers

**Published:** 1914-08-15

**Authors:** 


### THE IMPERIAL FORCES AND NATIONAL INSURANCE.

### The Position of Sailors and Soldiers.

The unfortunate war in which this country is now engaged brings into operation a subsection of the National Insurance Act of 1911, which was embodied in that measure to meet the circumstances of an emergency such as that which has now to be faced. By virtue of the subsection in question the Reserves of both forces of the Crown that have now been called out upon active service and the men of the Territorial force who have been embodied will be treated for the purpose of National Health Insurance as members of the Regular forces. Up to the present time we have considered the application of the National Insurance Acts to the naval and military forces of the Crown to be of interest to such a restricted number of our readers that we have refrained from any reference to the special features of the provisions that have been made for them under the Acts. The case is now quite altered.

Public interest in our sailors and soldiers is not confined to the actual operations of warfare, but extends to their personal welfare and that of their dependants, and not the least important of the matters to engage attention in this connection will be the position of the forces in regard to the scheme of National Insurance. It is therefore proposed to indicate briefly the special conditions affecting the insurance of men in the naval and military services of the Crown, whose numbers are immediately to be so largely increased.

### The Benefits and Contributions.

Owing to the fact that while a man is engaged in the naval or military service under the Crown he is provided with medical treatment and maintenance during illness as part of the emoluments of his employment there is no need for medical, sickness, or disablement benefit to be furnished for him from the National Insurance funds. In like manner if a member of either force becomes affected by tuberculosis while in the service he would be duly cared for; hence the provision of sanatorium benefit is equally unnecessary.

The only benefit conferred by the National Insurance Act which has no direct counterpart in the ordinary provision made for members of the services is maternity benefit. The maternity benefit is, however, one of the most beneficial of the advantages conferred by the Act, and it was therefore thought desirable to extend the privilege of participation therein to the members of the Regular forces. Moreover, although the benefits conferred by the Act are not required immediately while in the services it is necessary to make provision for the time when, upon leaving the colours, the men enter civil employment and thus become amenable to the ordinary requirements of their altered status in regard to Health Insurance.

Thus while serving in the Regular forces the only benefit actually receivable is maternity benefit, but the contributions payable are larger than the sum required to pay for this benefit by an amount which is sufficient to enable the men to enter insurance at the full rates of benefit on retirement irrespective of their then attained ages. The weekly contribution payable is 3d., of which 1½d. is deducted from the wage of the seaman, marine, or soldier, and the remaining 1½d. is payable out of funds provided by the Admiralty and the Army Council respectively.

The payment of contributions ceases on the termination of the period of his first engagement for any member of either force who has re-engaged for pension, unless he elects to continue the contributions with a view to providing the maternity allowances and for his entry into insurance upon his eventual retirement from the service. This option is allowed because it is presumed that the pension will be sufficient to obviate any dire distress should the man fall ill after his retirement. But as the pension is usually insufficient to provide for maintenance, and it is necessary to enter civil employment to supplement the pension the man will ordinarily be compelled to become insured. If he has not elected to continue his contributions during the period of re-engaged service he will enter insurance for benefits at the rate applicable to his then attained age. It is therefore generally advisable for the contributions to be continued.

### Soldiers and Approved Societies.

For the purpose of obtaining the maternity benefit the sailor or soldier may become a member of an approved society while he is in the service. He may, however, defer joining an approved society until his retirement. In this latter event the administration of the maternity benefit is in the hands of the Admiralty and Army Council, and is paid by them either directly or through Insurance Committees out of the Navy and Army Insurance Fund. It may be mentioned, in passing, that the maternity benefit is payable even though either or both the man and his wife are resident outside the United Kingdom at the date of the confinement and whether the man has joined an approved society or not. It may also be noted that the benefit is payable in respect of the birth of a posthumous child in precisely the same manner as though the father were living. There is an incidental advantage in joining an approved society while in the service at the earliest opportunity. This is so, because on retirement it may readily happen that the man's state of health will be such that no approved society will be willing to accept his application for membership. Having become a member of a society while in the service the society would be unable to refuse the right to continuation of membership, no matter what his state of health may be upon retirement. In order to obviate to some extent the disadvantages that might be expected to accrue to those men who, not having joined a society while in the service, are unable upon their discharge to secure membership in a society, it is provided that they shall be allowed to be readmitted to the Navy and Army Insurance Fund. By this means men so discharged are relieved of the necessity of becoming deposit contributors. The advantage thus conferred is very well exemplified by the figures given in the second annual report, which show that up to December 31 last out of 568 men who have been readmitted to the Fund only eighty-six have since been transferred to approved societies, leaving 482 on the Fund as at the end of the year. From the comparatively small number who have been readmitted no less than 282 have claimed sickness benefit, the amount so claimed being in total £2,118, or an average of fifteen weeks each at the rate of 10s. a week, while the report states that a considerable proportion have already exhausted their full twenty-six weeks' benefit. Much of this excess sickness is, no doubt, due to the fact that the men had become affected by tuberculosis, for 106 had been in receipt of sanatorium benefit.

### Seamen and Marines.

It is somewhat remarkable that a larger proportion of soldiers have become members of approved societies than is the case with seamen and marines. The second annual report states that out of the 337,000 men in the naval and military service of the Crown who are insured, 50,000 seamen and marines and 150,000 soldiers are known to have obtained admission to approved societies. In other words, the number of soldiers who have joined societies is three times as large as the number of seamen and marines. At the same time, the number of claims that have been paid in respect of maternity benefit out of the Navy and Army Insurance Fund —that is, for men who have not joined societies— were 2,713 in respect of seamen and marines and 1,100 in respect of soldiers.

### Effect of Insurance on Disabled Men.

By the calling up of the Reserves and the embodiment of the Territorials, together with the enlistment of a large number of recruits, there will be a large transfer from the class of ordinary employed contributors to the Navy and Army class of insured persons. To some extent this may mean a relief to the insurance funds, because it is unfortunately extremely probable that the risks of warfare will add to the death-roll more than the normal proportion of the insured population. Unlike life assurance, a heavy mortality experience means, in general, a reduction of liability in sickness insurance. Furthermore, owing to the provision that sickness benefit is only payable to the amount of the excess of the ordinary rate over the amount of any compensation that may be receivable in the case of compensated damage, the charge on the approved societies for disabled sailors and soldiers after they have retired will probably be little, if any, more than the normal liability for insured persons in time of peace. The actual effects cannot be foretold with any degree of certainty. One thing at least, however, appears to be clear, and that is that National Health Insurance should materially assist to avert the distressing circumstances which in the past have frequently compelled disabled sailors and soldiers to solicit public alms.

### The Position of Nurses.

There is a matter which would seem to call for attention by the Insurance Commissioners, and that is the insurance of nurses attending the forces. The nurses will in most cases be already insured, but it does not seem to be right that they should be required to pay the full ordinary contribution while they are attending the forces. They are in effect, if not actually, in the service of the Crown, and as such they will presumably receive not only medical treatment and attendance, but will also be furnished with maintenance if they should themselves unfortunately fall ill. In consequence, there is for them, at least temporarily, no necessity for Health Insurance, and effect should be given to this in the contributions payable. We do not recollect any provision of the Acts which contemplates in any way the present circumstances in which such nurses are placed, and an early decision of the Insurance Commissioners is desirable.

